# Impedance Spectroscopy of Sm-Doped of BaBi_2_Nb_2_O_9_ Aurivillius Ceramics

**DOI:** 10.3390/ma17174360

**Published:** 2024-09-03

**Authors:** Jolanta Makowska, Michał Rerak, Beata Wodecka-Duś, Tomasz Goryczka, Grzegorz Tytko, Anna Zawada, Małgorzata Adamczyk-Habrajska

**Affiliations:** 1 Institute of Materials Engineering, Faculty of Science and Technology, University of Silesia, 75 Pułku Piechoty 1A, 41-500 Chorzow, Poland; jolanta.makowska@us.edu.pl (J.M.); michal.jan.rerak@gmail.com (M.R.); beata.wodecka-dus@us.edu.pl (B.W.-D.); tomasz.goryczka@us.edu.pl (T.G.); 2 Faculty of Automatic Control, Electronics and Computer Science, Silesian University of Technology, 44-100 Gliwice, Poland; grzegorz.tytko@polsl.pl; 3 Department of Materials Engineering, The Czestochowa University of Technology, Armii Krajowej 19, 42-200 Czestochowa, Poland; anna.zawada@pcz.pl

**Keywords:** impedance spectroscopy, BaBi_2_Nb_2_O_9_, ceramics, Aurivillius structure, Sm^3+^

## Abstract

This investigation focuses on the impact of Sm^3+^ dopants on BaBi_2_Nb_2_O_9_ (BBN) ceramics. These ceramics were obtained using the traditional solid state reaction approach. Techniques like scanning electron microscopy (SEM) and energy dispersive X-ray spectroscopy (EDS) were employed to explore the structure and morphology of the ceramics. The results showed that the chemical composition of the ceramic samples matched well with the initial ceramic powder stoichiometry. Increasing the amount of samarium resulted in a slight reduction in the average ceramic grain size. The ceramics exhibited a tetragonal structure categorized under the space group I4/mmm. The electrical properties were analyzed using complex impedance spectroscopy (SI) across various temperatures and frequencies, revealing that both grains and intergranular boundaries are significant in the material’s conductivity.

## 1. Introduction

Materials science has seen growing interest in exploring and developing advanced ceramic materials to enhance the performance and sustainability of various technological applications. This trend has been extensively documented in the recent literature [1,2,3]. Concurrently, there has been a significant push toward developing environmentally friendly materials, specifically those devoid of lead, due to the environmental and health concerns associated with its use [4,5,6]. There exists a certain range of materials with a perovskite-type structure which can be described by the general formula ABX_3_, where the position of atom A is occupied by ions of alkali metals or rare earth metals while the position of atom B is filled by ions of transition metals [7,8,9,10]. The undisturbed, theoretical crystal structure of perovskite consists of two pyramids with a common base situated at the center of a cube. The corners of the cube are occupied by A atoms, and in the center lies the B atom. The B atom is surrounded by six oxygen atoms located in the middle of each face of the cube. The oxygen atoms, together with the B atom, form a BO_6_ octahedron [11]. The perovskite structure is the starting form for the construction of compounds with a layered perovskite structure, among which Aurivilius-type phases can be distinguished [12,13,14,15,16,17]. Compounds with the Aurivillius structure are composed of regularly arranged layers (Bi_2_O_2_)^2+^ and perovskite blocks (A*_n_*_−1_B*_n_*O_3*m*+1_)^2−^, where *n* is the number of layers of these blocks. Sublattice A is typically occupied by cations such as Bi^3+^, Sr^2+^, Ba^2+^, and Ca^2+^ [18,19]. Meanwhile, in sublattice B, ions such as Nb^5+^, Ta^5+^, W^6+^, and Mo^6+^ are found [19,20]. Numerous possibilities for ion substitutions both in the perovskite block structure and in the bismuth-oxygen layers create a broad spectrum of modifications for the compounds discussed and, consequently, almost arbitrary modifications of their physical properties.

In this context, BaBi_2_Nb_2_O_9_ (BBN) ceramics have emerged as a particularly promising class of materials [21,22,23,24,25,26,27]. Extensive studies have highlighted their potential applications and advantages, making them an important point of current research [13,28,29,30,31]. BaBi_2_Nb_2_O_9_ ceramics crystallize in an orthorhombic or tetragonal structure [13,32,33,34,35] and show a broadened ferroelectric-paraelectric transition at *T* = 100–150 °C with a relaxor characteristic [35]. This compound has garnered significant interest, owing to its diverse utility in high-temperature piezoelectric materials and non-volatile random-access memory (NVRAM), which are primarily attributable to its fatigue-resistant characteristics [31,36]. To optimize their performance for specific properties, it is essential to tailor their characteristics through dopant incorporation [36,37].

The objective of this research was to investigate how Sm^3+^ doping affects the impedance characteristics of BaBi_2_Nb_2_O_9_ Aurivillius ceramics. Utilizing extensive impedance spectroscopy analysis encompassing a diverse spectrum of temperatures and frequencies, this research endeavors to delineate the underlying mechanisms through which samarium doping modulates the electrical properties of these ceramic materials. Understanding these mechanisms is crucial for optimizing the material’s performance for specific applications and the development of new materials with tailored electrical properties.

## 2. Materials and Methods

The research material was produced through conventional solid phase synthesis and free sintering in an air atmosphere, beginning with the precise weighing of substrates such as barium carbonate (BaCO_3_), bismuth (III) oxide (Bi_2_O_3_), niobium (V) oxide (Nb_2_O_5_), and samarium (III) oxide (Sm_2_O_3_) in stoichiometric quantities. The oxides and carbonates, in stoichiometric ratios, were milled in a planetary ball mill for *t* = 24 h using 97% ethyl alcohol (POCH CZDA), followed by air-drying of the homogenized mixture for *t* = 48 h. Subsequently, the mixture was molded into cylindrical disks with a diameter of 25 mm using a hydraulic press set at *p* = 300 MPa. The synthesis was carried out at *T* = 950 °C for *t* = 4 h using the solid phase synthesis method. After synthesis, the compacted materials were pulverized in a mortar, wet-milled for another *t* = 24 h, and subsequently air-dried for *t* = 48 h. The resulting ceramic powder was then compressed into *d* = 10 mm diameter discs at *p* = 600 MPa using a hydraulic press. Finally, these prepared materials underwent free sintering in an air environment at *T* = 1100 °C for *t* = 2 h.

The microstructures and chemical compositions of the resulting ceramics were examined using a JSM-7100F scanning electron microscope (SEM) coupled with a NORAN Vantage energy dispersive spectrometer (EDS). Sample image registration followed a procedure involving randomly selecting multiple fields distributed across the entire surface of the ceramic samples under testing. Qualitative and quantitative chemical composition assessments were performed using the X-ray microanalysis method.

The phase analysis and structural characterization of the examined material were conducted using the X”Pert PRO X-ray diffractometer manufactured by PANAlytical. The experimental diffraction pattern’s intensities and positions of diffraction lines were cross-referenced with the international ICDD-PDF4 database (JCPDS card no. 00-40-0355).

For impedance spectroscopy testing, the set-up included a Hewlett-Packard 4192A impedance analyzer, a Hewlett-Packard 34401A millivoltmeter, and a Shimaden FP93 temperature controller. Impedance measurements were carried out over a temperature range of *T* = 500–823 K in increments of *T* = 10 K and a frequency range from *f* = 20 Hz to *f* = 2 MHz. Before data analysis, a preliminary test was conducted each time to verify data consistency using the Kramers–Kroning relations.

## 3. Results

### 3.1. X-ray Diffraction

**Figure 1:**
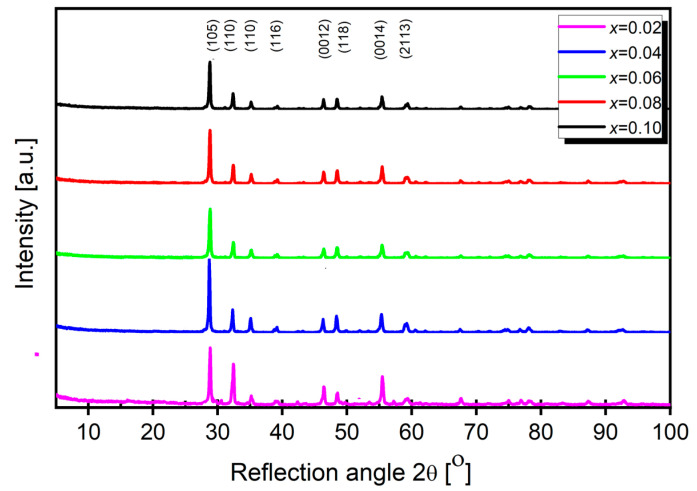
X-ray diffraction pattern of BBN ceramics doped with Sm^3+^ ions for various modifier concentrations.

XRD measurements were conducted to confirm the single-phase nature of the sample and to investigate the effect of samarium doping on the unit cell parameters. The obtained diffraction patterns are presented in Figure 1.

Phase identification was the first step in analyzing the obtained diffraction spectra. The intensity and position of the diffraction lines from the experimental diffractogram were compared to the standard from the international ICDD database. It was found that the BaBi_2_Nb_2_O_9_ phase was the only one present in the sample (JCPDS card no 00-12-0403). The study indicated that the ceramic materials under consideration were of a single phase at room temperature. A detailed analysis of the obtained diffractograms showed that the substitution of Sm^3+^ caused a slight shift in the diffraction peak position toward lower angles and broadening of the diffraction peaks. For the peaks in the diffractograms of ceramics with the minimum and maximum dopant concentrations, this shift was 0.124°. All the materials discussed maintained a tetragonal phase characterized by the I4/mmm space group, regardless of the dopant ion concentration. In the next stage of the X-ray test result analysis, the unit cell parameters and the unit cell volume (V) were determined using the Rietveld method [38] based on the obtained X-ray spectra. The values of the crystallographic parameters are listed in Table 1.

A detailed analysis of the obtained diffractograms showed that the substitution of Sm^3+^ caused a slight impact on the crystal lattice. This can be clarified as follows. The ionic radius of samarium is $r_{{\mathrm{Sm}}^{3+}}={0.93\cdot 10}^{-10}m$, while the ionic radius of barium is $r_{{\mathrm{Ba}}^{2+}}$ = 1.36·10^−10^ m, and bismuth’s is $r_{{\mathrm{Bi}}^{3+}}$ = 0.75·10^−10^ m [39]. Thus, it would be expected that changes in network constants will be significant. However, the data analysis in Table 1 indicates that the unit cell parameters gradually decreased, which can be explained by the difference in the samarium and barium ionic radii. This fact confirms the thesis by Y. Wu et al. [36] based on studies of BaBi_2_Nb_2_O_9_ ceramics modified with vanadium. According to them, the specific structure of layered perovskites, particularly their presence in their structure of bismuth-oxygen layers, prevents drastic changes in the network.

### 3.2. Microstructure and EDS Analysis

**Figure 2:**
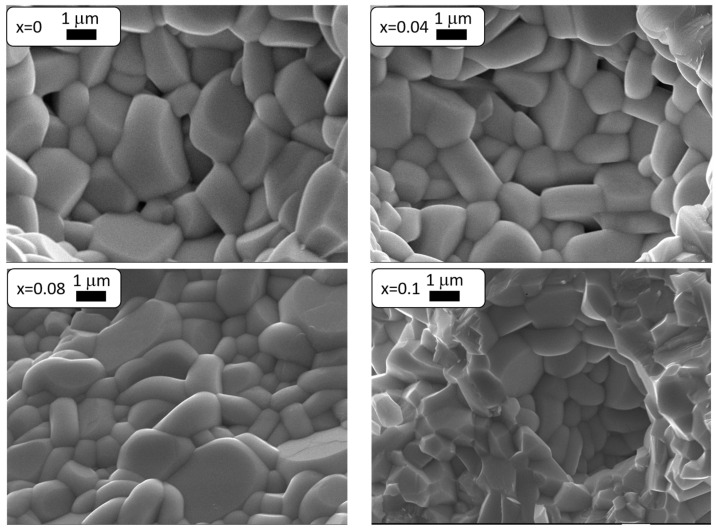
SEM image of BaBi_2_Nb_2_O_9_ ceramics doped with Sm^3+^ for selected dopant concentrations.

Figure 2 presents SEM images of BaBi_2−*x*_Sm*_x_*Nb_2_O_9_ ceramic fractures for the selected concentrations of added samarium admixture.

**Figure 3:**
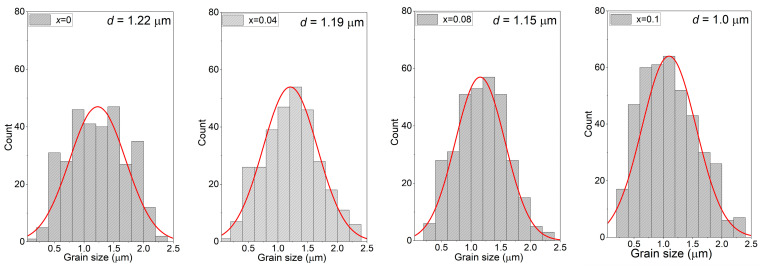
Grain size distribution diagrams.

The analysis of SEM images of BaBi_2_Nb_2_O_9_ ceramics doped with samarium ions allowed us to notice that an increase in the concentration of the dopant (Sm^3+^) had a beneficial effect on the microstructure of the tested material. It became more fine-grained and homogeneous, increasing the number of better-developed grains. Visible angular shapes became rounded with a growing concentration of samarium admixture in the material. Additionally, the third dimension (grain thickness) was much smaller than the other two, which is a characteristic feature of the Aurivillius structure and is related to the fact that the force forcing the crystallites to grow along the c axis, perpendicular to the plate surface, is much smaller than the forces acting along the a and b axes [40]. The authors of [25] observed a similar relationship between the increase in the modifier concentration and the dimensions and shape of the grains. Figure 3 shows the grain size distribution diagrams, which were made based on the analysis of several photos for a given concentration with the same magnification. When analyzing the obtained results, it can be stated that with the increase in the Sm^3+^ dopant concentration, the grain size decreased from *d* = 1.22 for *x* = 0 to *d* = 1.0 for *x* = 0.1.

EDS analysis allowed us to determine the chemical elements’ qualitative and quantitative compositions constituting the tested material. The quantitative analysis of the chemical compositions was carried out in randomly selected micro-areas of each of the considered ceramic materials. Based on the obtained test results, it was found that the dispersion between the average percentage contents of individual components of the discussed compounds and the assumed theoretical stoichiometry was small and within the error limits of the method used (Table 2).

### 3.3. Dielectric Measurements

**Figure 4:**
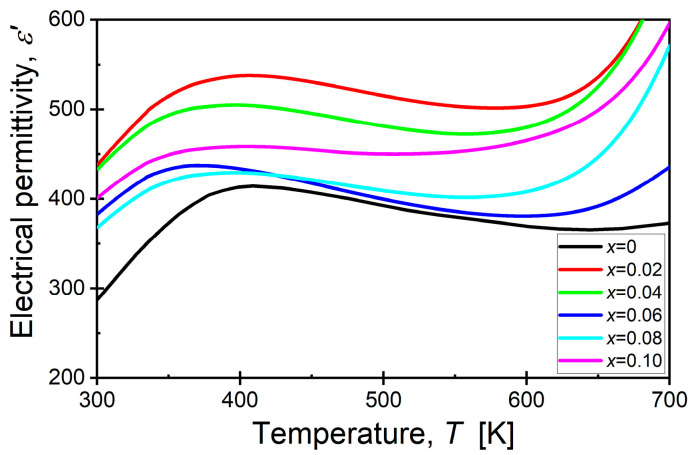
Temperature dependence of the real part of electric permittivity, BaBi_2_Nb_2_O_9_ ceramics modified with Sm^3+^ ions obtained for a frequency of measuring field equal to 1 kHz.

Before conducting the key impedance measurements for this publication, we also performed measurements of the temperature dependence of the dielectric permittivity. The characteristics obtained at a measurement field frequency of 1 kHz are presented in Figure 4. The samarium dopant caused a significant increase in the dielectric permittivity values across the entire temperature range studied compared with the unmodified materials. Additionally, the temperature at which the maximum dielectric permittivity occurred shifted to lower temperatures. A more detailed description of the properties is provided in a second publication, which includes a thorough analysis of the dielectric properties.

### 3.4. Impedance Spectroscopic Studies

It is widely known that the electrical properties of ceramics are a composite of contributions from various elements of the material’s microstructure, as well as the processes occurring within them. Charge transport can occur through mechanisms such as charge displacement, dipole reorientation, and the involvement of so-called space charge associated with structural inhomogeneity (i.e., defects, impurities, etc.) [41,42]. In the presence of an applied alternating electric field, dielectric relaxation is observed, caused by various types of polarization correlated with charge transport processes. The interrelation between the electrical properties and the microstructure of ceramic material can be experimentally studied using a highly convenient and powerful tool, namely impedance spectroscopy. Conducting impedance measurements over a wide range of measurement field frequencies allows for the separation of contributions from different electroactive regions (grains and grain boundaries, as well as electrode-adjacent layers) to the conduction process characteristic of the material under study. Impedance spectroscopy describes the electrical processes occurring in ceramics upon the application of an AC signal as an input disturbance.

The obtained results for the impedance spectroscopy measurements of the discussed ceramics are presented in the form of plots illustrating the dependence of the real and imaginary parts of impedance on the frequency of the input signal.

**Figure 5:**
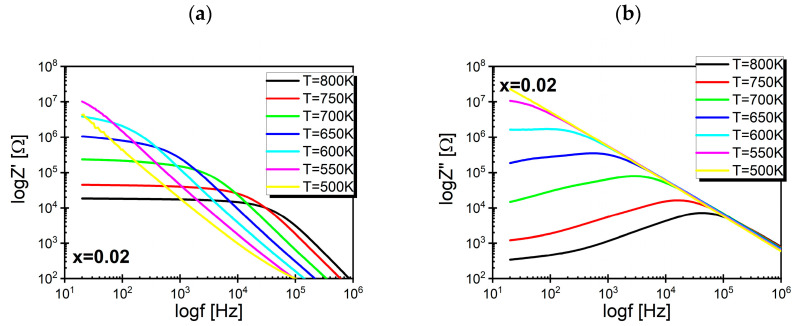

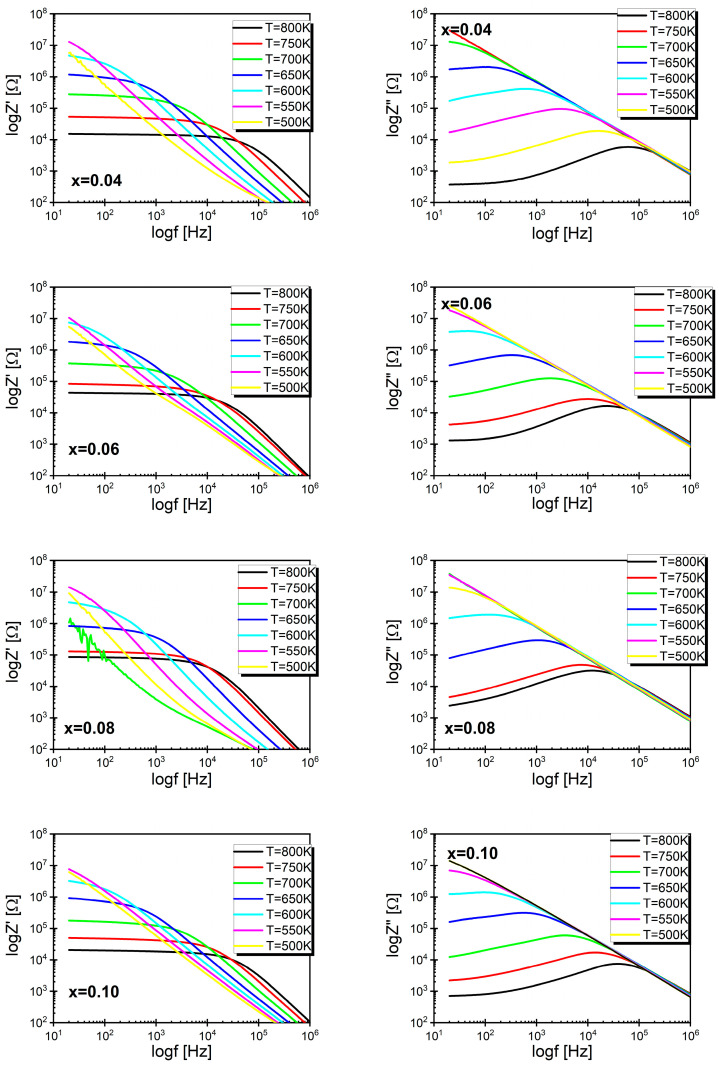
(**a**) A set of graphs showing the real part of impedance as a function of the frequency. (**b**) A set of graphs presenting the frequency characteristics of the imaginary part of impedance for BaBi_2_Nb_2_O_9_ ceramics modified with samarium ions substituted into the bismuth-oxygen layers of the Aurivillius structure, measured at selected temperatures in the range of T = 500–823 K.

The curves describing the frequency dependence of the real part of the impedance can be divided into two regions. The first region in the low-frequency range is where the value of Z′ is practically independent of the frequency, whereas the second region is the area of strong dispersion of Z′. The dependencies presented in Figure 5a indicate that as the temperature increased, the first region expanded significantly at the expense of the second region.

The measurement points describing the dependence of log*Z*″(logf) formed a curve which reached a maximum at a frequency f_max_. The position of this maximum on the frequency axis depends on the temperature; as the temperature rises, it shifts toward higher frequencies. This behavior is characteristic of materials whose microstructures consist of grains and grain boundaries, but the active regions are the grain boundaries, which become a reservoir of space charge. The mobility of these charge carriers increases with rising temperatures, which reduces the relaxation time of the mobile charges and causes the shift of the frequency f_max_ toward higher values. The strong temperature dependence is also exhibited by the maximum value of the imaginary part of the impedance (Z″_max_). Its decrease can be correlated with temperature changes in the material resistance (Figure 5b). Merging of the logZ″(logf) characteristics into a single curve in the high-frequency range indicates significant space charge accumulation [43,44]. Even a cursory analysis of the logZ″(logf) dependency reveals that the half-width of the curves exceeded 1.2 decades, indicating deviations in the relaxation processes in the discussed materials from ideal Debye behavior [45]. However, this raises questions about the temperature dependence of the half-width of Z″(f) curves. To answer this question, normalization of the curves must be performed. The frequency f_max_ is calculated from the following condition:

(1) $$\frac{{dZ}^{\prime \prime }}{df}\left(f_{max}\right)=0$$

**Figure 6:**
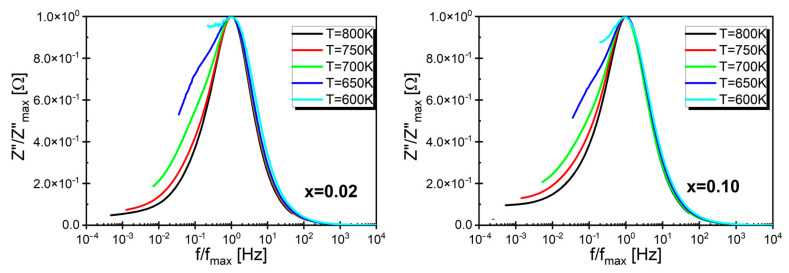
The normalized imaginary part of the impedance Z″/Z″_max_ of BaBi_2_Nb_2_O_9_ ceramic doped with samarium ions for mole fractions *x* = 0.02 and *x* = 0.10, presented as a function of the normalized frequency f/f_max_.

Examples of the obtained normalized curves are presented in Figure 6 (the rest of the characteristics can be found in Figure S1 of the Supplementary Materials). The presented curves coincide in the frequency range above f_max_, indicating that the nature of the dependency remained unchanged with temperature variations, and according to the assumptions of the authors of [46,47], it may be associated with a significant contribution of the aforementioned spatial charge in the sample volume. However, in the frequency range below f_max_, a strong temperature dependence of the curve shape was observed.

**Figure 7:**
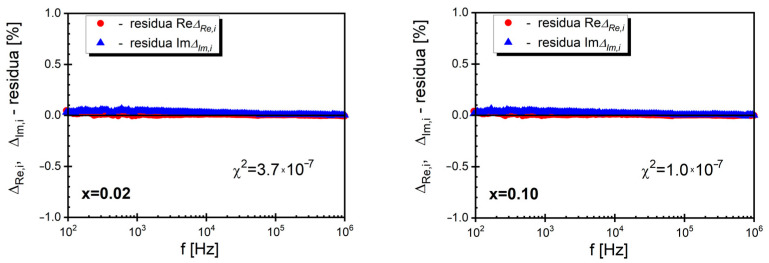
The frequency dependence of the residuals of the real and imaginary parts of the impedance of BaBi_2_Nb_2_O_9_ ceramics doped with samarium for mole fractions *x* = 0.02 and *x* = 0.10.

The consistency of the obtained data was confirmed using K-K test V1.01 based on the Kramers–Kronig method [48,49], which was widely described in our previous paper [50]. All obtained experimental data were subjected to the mentioned test, and an example of the frequency-dependent calculated residuals is included in Figure 7.

The values of the obtained residuals for both the real and imaginary parts of the impedance spectrum did not exceed 2%, and the value of χ^2^ was not greater than 6·10^−5^. Additionally, it should be noted that in the curves presented in Figure 6, as well as in the case of other curves, the residuals were randomly distributed around the frequency axis. This fact provides further confirmation of the consistency of the data and indicates the justification for further analysis.

**Figure 8:**
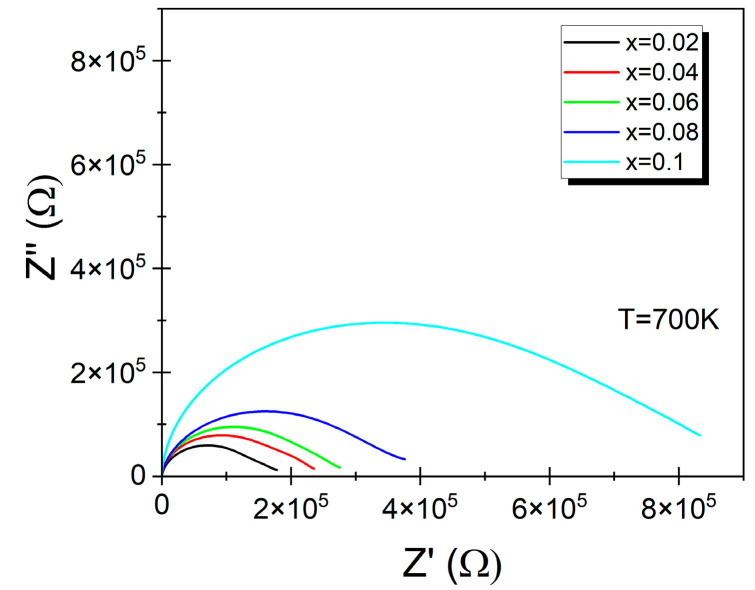
Nyquist plots (Z″ vs. Z′) of BaBi_2−*x*_Sm_*x*_Nb_2_O_9_ ceramics at temperature T = 700 K.

Finding the appropriate equivalent circuit would significantly facilitate the plotting of Nyquist plots. An example of this type of plot, showing the dependence of the imaginary part as a function of the real part of the impedance for all discussed samples at a temperature T = 700 K, is presented in Figure 8.

The presented dependencies took the form of deformed yet symmetrical circles, with their centers located below the axis representing the real part of the impedance. The angle is between the tangent and the *Z*″(*Z*′) curve at frequencies *f* → ∞, and the real axis is 90°, which is characteristic of a Debye-type response [45]. However, at low frequencies (*f* → 0), the tangents of the circles formed an angle smaller than 90°. Moreover, the value of this angle also depends on the temperature. These observations suggest that the presented dependencies resulted from the superposition of two semicircles, representing the response from two components of the microstructure: grains and grain boundaries [13,51].

**Figure 9:**
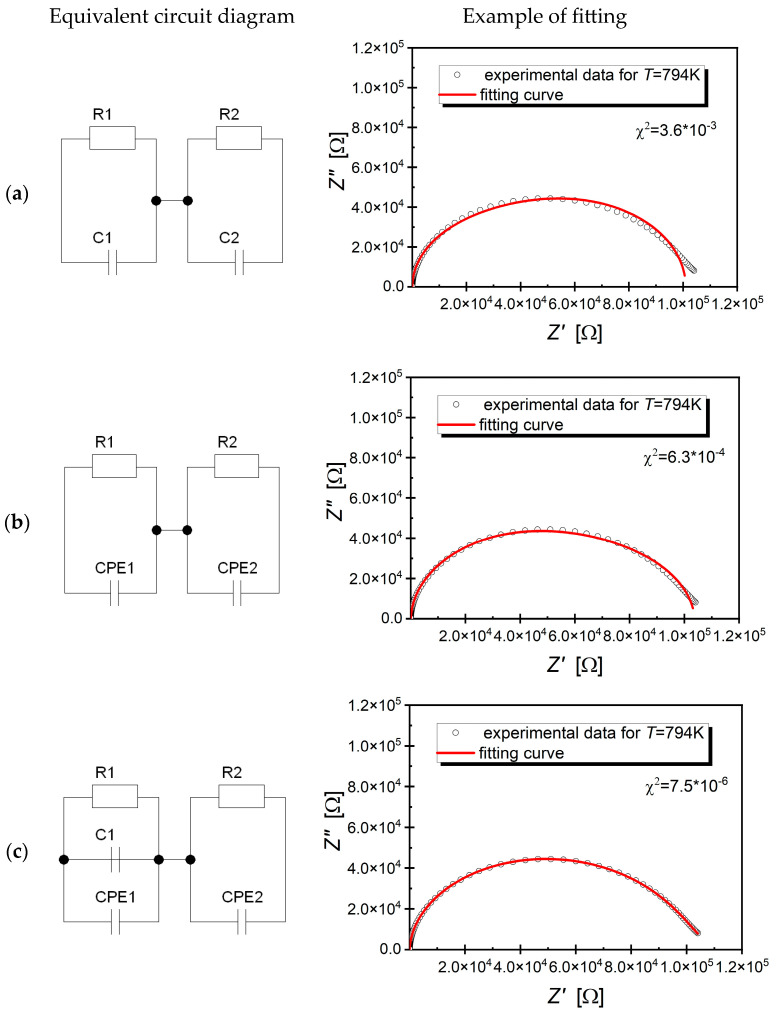
(**a**–**c**) Considered equivalent circuit models describing the conduction phenomena and an example of data fitting.

Typically, Nyquist plots of this type are described by an equivalent circuit composed of two Voigt elements connected in series. In the case of the data presented in this study, the use of this circuit did not yield satisfactory results. The value of the χ^2^ parameter was too high (Figure 9a). In such cases, replacing the capacitance with a constant phase element (CPE) is standard practice. This approach significantly improved the fit quality, but it remained unsatisfactory (Figure 9b). Based on the literature data, a decision was made to add a capacitive element to the branch of the circuit describing the properties of the grains. Such equivalent circuits are often used to describe the electrical properties of ceramic materials [52,53]. They result from adopting the Bauerle model [54,55]. In this model, it is assumed that in real ceramic materials, the grain boundaries disappear in some places, and the grain interiors come into contact. If the conductivity of the grains is higher than the conductivity of the grain boundaries, then the points of contact between the grains become pathways for rapid ion transport [56]. The proposed change brought positive effects in the form of a significant improvement in the fit quality (Figure 9c); the χ^2^ parameter took on an extremely low value, which confirmed the good fit quality.

**Figure 10:**
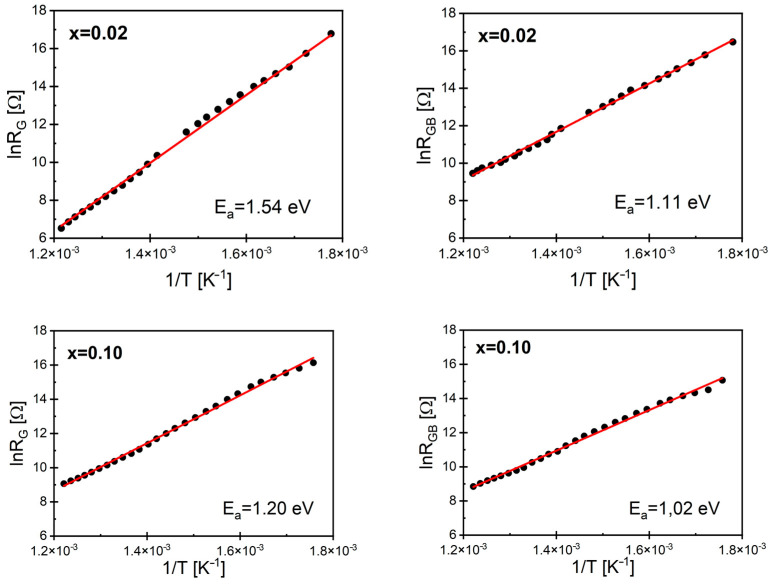
Dependence of the lnR_G_(1/T) and lnR_GB_(1/T) of BaBi_2_Nb_2_O_9_ ceramics doped with samarium for mole fractions *x* = 0.02 and *x* = 0.10.

The obtained grain and grain boundary resistances are presented as lnR(1/T) graphs in Figure 10 for samarium ion-doped BBN ceramics with *x* = 0.02 and *x* = 0.1. (The rest of the characteristics can be found in Figure S2 in the Supplementary Materials).

The value of the grain resistance was greater than the resistance of the grain boundary regions, which is a consequence of the fact that grain boundaries serve as a reservoir of space charge. It is also worth noting that these differences became more pronounced as the temperature decreased. The linear nature of the presented relationships confirms the activation form of the conduction processes. Based on the commonly known Arrhenius dependence, the activation energy of the conduction processes occurring in the grains (*E*_G_) and grain boundaries (*E*_GB_) was determined (Table 3).

The value of the activation energy of the AC conduction process in the grain boundaries showed an increasing trend with the increasing content of samarium ions Sm^3+^ compared with undoped ceramics. However, this increase was not uniform. Additionally, it is noteworthy that there were significant differences between the activation energy of grains and that of the grain boundaries.

### 3.5. AC Conductivity

**Figure 11:**
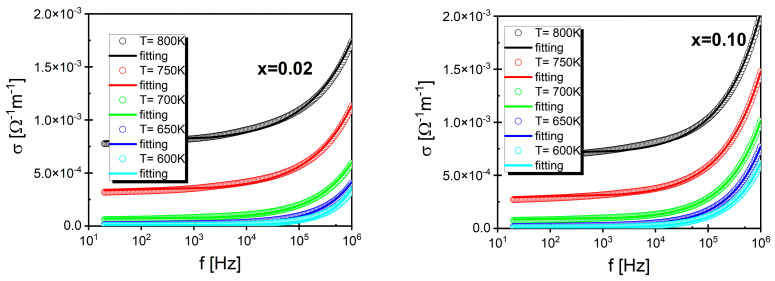
Frequency dependence of the conductivity of BaBi_2_Nb_2_O_9_ ceramics doped with samarium for mole fractions *x* = 0.02 and *x* = 0.10.

Using complex impedance measurements, the electrical conductivity σ was calculated with the following equation:

(2) $$\;\sigma =\frac{d}{A}\frac{Z^{\prime }}{\left|Z^{*}\right|}$$

where d and A represent the length and the transversal sectional area of the sample and $Z^{*}=\sqrt{Z^{\prime 2}+{Z\prime \prime }^{2}}$ is the complex impedance module of the sample. Examples of the obtained electrical conductivity frequency characteristics at selected temperatures for ceramics with *x* = 0.02 and *x* = 0.1 are shown in Figure 11 (The rest of the characteristics can be found in Figure S3 in the Supplementary Materials.).

In the presented *σ*(f) characteristics, two regions can be distinguished. The first one occurred in the low-frequency range and had the form of an f-independent plateau. This region was associated with the *DC* component of electric conductivity (*σ*_DC_). On the other hand, the second region, associated with the *AC* component of electric conductivity (*σ*_AC_), was characterized by a strong frequency dependence. Thus, the electric conductivity in the discussed ceramic materials is the sum of DC and AC conductivity, which can be expressed in the form of the following relationship:

(3) $$\sigma \left(f\right)=\sigma_{DC}\left(T\right)+\sigma_{AC}\left(f,T\right)$$

**Figure 12:**
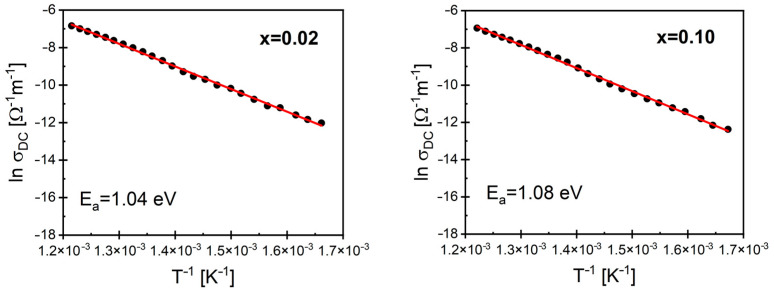
The temperature dependence of the DC conductivity of BaBi_2_Nb_2_O_9_ ceramics doped with Sm^3+^ ions to the amounts of *x* = 0.2 and *x* = 0.1.

**Figure 13:**
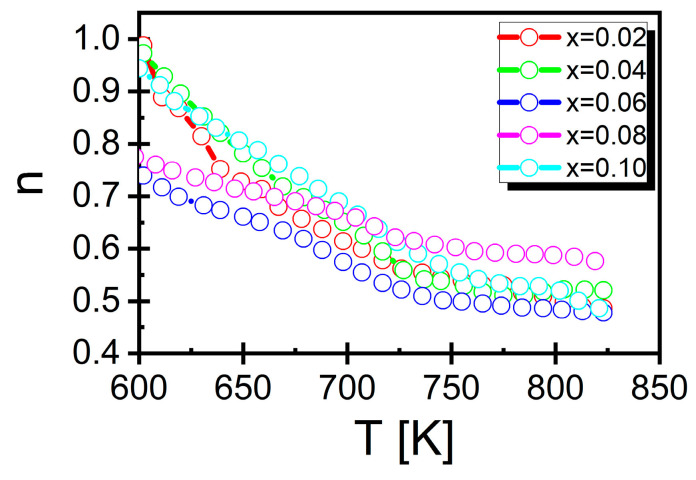
Temperature dependence of the exponent n (Jonscher’s law) for BaBi_2_Nb_2_O_9_ ceramics doped with Sm^3+^ ions.

The presented dependencies show the same character as the conductivity for a wide range of ceramic materials [58,59,60,61,62], which suggests that the function *σ*(f) can be modeled by Jonscher’s law [63]:

(4) $$\sigma \left(\omega \right)=\sigma_{DC}+A\omega^{n}$$

where ω=2πf, σ_DC_ is the DC conductivity corresponding to the frequency-independent part of *σ*(f), n is the power law exponent (0 ≤ n ≤ 1), and A is the pre-exponential factor. This relationship held true in the low-frequency regime (i.e., below 100 MHz). Jonscher’s law was fitted to the experimental data, which allowed for the determination of the temperature dependence of σ_DC_, (Figure 12 shows an example for mole fractions *x* = 0.02 and *x* = 0.10. The rest of the characteristics can be found in Figure S4 in the Supplementary Materials) conductivity, and the coefficient n (Figure 13).

The value of σ_DC_ increased with rising temperatures, which may suggest an activation character of the conductivity. This thesis was confirmed by the linear character of the lnσ_DC_(1/T) dependence (see Figure 12). For low concentrations of the samarium dopant, the activation energy determined from the Arrhenius dependence had a low value, which showed a slight increasing trend with the rise in dopant content. Based on the results presented in this work, as well as those in [64], it can be concluded that the base compound BaBi_2_Nb_2_O_9_, like many other Aurivillius compounds, is characterized by high charge carrier mobility. The substitution of samarium ions slightly distorts the crystal lattice, which in turn leads to the scattering of charge carriers and a reduction in conductivity [64]. On the other hand, samarium ions substituted into the bismuth-oxygen layers act as a heterovalent dopant, which is associated with the creation of oxygen vacancies. This should contribute to an increase in conductivity within the material. The existence of these two mutually exclusive processes results in nonuniform changes in activation energy observed in both the grains and grain boundaries.

Apart from the temperature behavior of direct current conductivity, the exponent n in Jonscher’s law (Equation (4)) is also noteworthy (Figure 12). It depends on the temperature, and the shape of this dependence plays a key role in determining the mechanism of electrical conductivity in a given material.

Several models related to the temperature behavior of the exponent n can be found in the literature. The first one is the so-called quantum mechanical tunneling (QTM) model. The assumptions of this model were widely discussed by the authors of article [63]. For the purposes of this work, it is worth mentioning that within this model, the value of the n coefficient is close to 0.8 and is practically independent of the temperature. Another model describing the temperature behavior of the exponent n is the overlapping large polaron tunneling (OLTP) model. In this model, the discussed exponent n depends on both the frequency and temperature. As the temperature increases, its value initially decreases, reaches a minimum, and then increases [65]. In turn, a constant increase in the exponent n with increasing temperatures is predicted by the non-overlapping small polaron (NSPT) model [66], while its decrease is described by the correlated barrier hopping (CBH) model [65,67]. In the case of BBN ceramics doped with samarium-modifying bismuth-oxide layers, the value of the n factor decreased with increasing temperatures (Figure 13), which means that the hopping conductivity, according to the CBH model, was due to the reduction in the effective potential barrier caused by the overlapping of Coulomb potential wells. This situation significantly facilitates the hopping of a single electron between neighboring sites [68,69].

## 4. Conclusions

Aurivillius phase ceramics with the composition BaBi_2−*x*_Sm*_x_*Nb_2_O_9_, with *x* varying from 0 to 0.1 in increments of 0.02, were synthesized through solid phase synthesis and subsequently sintered freely in air. When analyzing the microstructure of the obtained ceramics, it was observed that increasing the samarium content resulted in a slight reduction in the average grain size of the ceramics. Both the pure and doped ceramics exhibited a tetragonal crystal structure with the space group I4/mmm, indicating that the doping did not alter the fundamental symmetry of the material. The electrical properties of the ceramics were thoroughly examined using complex impedance spectroscopy across a wide range of temperatures and frequencies. The analysis revealed that both the individual grains and the interfaces between them (grain boundaries) played significant roles in the material’s conductivity. The electrical conductivity in these materials was due to hopping. The shape of the temperature dependence of the exponent n indicates that the single electrons moved between neighboring sites. Based on the observed increase in activation energy for both DC conductivity (determined by fitting Jonscher’s law) and the conductivity of grains and grain boundaries, it can be assumed that the aforementioned movement of electric charges was somewhat hindered in the case of the doped materials. The reduced mobility of charges was a result of changes in the crystal lattice caused by the substitution of samarium ions in place of bismuth ions in the bismuth-oxygen layers. The reduction in conductivity led to the observed increase in the dielectric constant of doped materials and translated into enhanced application potential for the discussed materials.

## Figures and Tables

**Table 1:** Results of X-ray structural analysis of BaBi_2_Nb_2_O_9_ ceramics doped with samarium ions.

*x* (Sm)	BaBi_2−_*_x_*Sm*_x_*Nb_2_O_9_			
	*a* (Å)	*b* (Å)	*c* (Å)	*V* (Å^3^)
0.00 [32]	3.9406	3.9406	25.6378	398.1
0.02	3.9303 (1)	3.9303 (1)	25.6248 (3)	395.8 (3)
0.04	3.9291 (3)	3.9291 (3)	25.6127 (4)	395.4 (1)
0.06	3.9285 (2)	3.9285 (2)	25.6089 (4)	395.2 (3)
0.08	3.9259 (4)	3.9259 (4)	25.5957 (2)	394.5 (1)
0.10	3.9285 (1)	3.9285 (1)	25.6059 (3)	395.1 (8)

**Table 2:** Theoretical and experimental summary of substrate content for BaBi_2−x_Sm_x_Nb_2_O_9_ ceramics, expressed as oxides.

x	Theoretical Substrate Content (%)				Content of Substrates from EDS (%)			
	BaO	Bi_2_O_3_	Nb_2_O_5_	Sm_2_O_3_	BaO	Bi_2_O_3_	Nb_2_O_5_	Sm_2_O_3_
0.02	22.3	52.2	30.1	0.4	20	50.6	29.1	0.3
0.04	22.4	51.7	30.1	0.8	21	50.4	27.8	0.8
0.06	22.4	51.3	30.2	1.2	23.2	46.9	28.9	1.1
0.08	22.4	50.8	30.2	1.6	21.7	45.8	31.1	1.4
0.10	22.4	50.3	30.2	2.0	17.6	50.5	29.7	2.1

**Table 3:** The activation energy values of the conduction process in the grains and grain boundaries of BaBi_2_Nb_2_O_9_ ceramics modified with samarium ions.

*x* % Sm	*E_G_* (eV)	*E_GB_* (eV)
0.00 [57]	1	0.98
0.02	1.54	1.11
0.04	1.08	1.36
0.06	1.22	1.25
0.08	0.86	1.23
0.10	1.22	1.02

## Data Availability

Data are contained within the article.

## Supplementary Materials

The following supporting information can be downloaded at: https://www.mdpi.com/article/10.3390/ma17174360/s1, Figure S1: The normalized imaginary part of the impedance Z″/Z″_max_ of BaBi_2_Nb_2_O_9_ ceramic doped with samarium ions for mole fractions *x* = 0.04, *x* = 0.06 and *x* = 0.08, presented as a function of the normalized frequency f/f_max_; Figure S2: Dependence of the lnR_G_(1/T) and lnR_GB_(1/T) of BaBi_2_Nb_2_O_9_ ceramics doped with samarium for mole fractions *x* = 0.04, *x* = 0.06 and *x* = 0.08; Figure S3: Frequency dependence of the conductivity of BaBi_2_Nb_2_O_9_ ceramics doped with samarium for mole fractions *x* = 0. 0.04, *x* = 0.06 and *x* = 0.08; Figure S4: The temperature dependence of the DC conductivity of BaBi_2_Nb_2_O_9_ ceramics doped with Sm^3+^ ions to the amounts of *x* = 0.04, *x* = 0.06 and *x* = 0.08.
