# Supplementary figures and images for: Impedance Spectroscopy of Sm-Doped of BaBi2Nb2O9 Aurivillius Ceramics
Source: Materials (Basel). 2024 Sep 3;17(17):4360. doi: 10.3390/ma17174360 (PMC11396611; doi:10.3390/ma17174360)

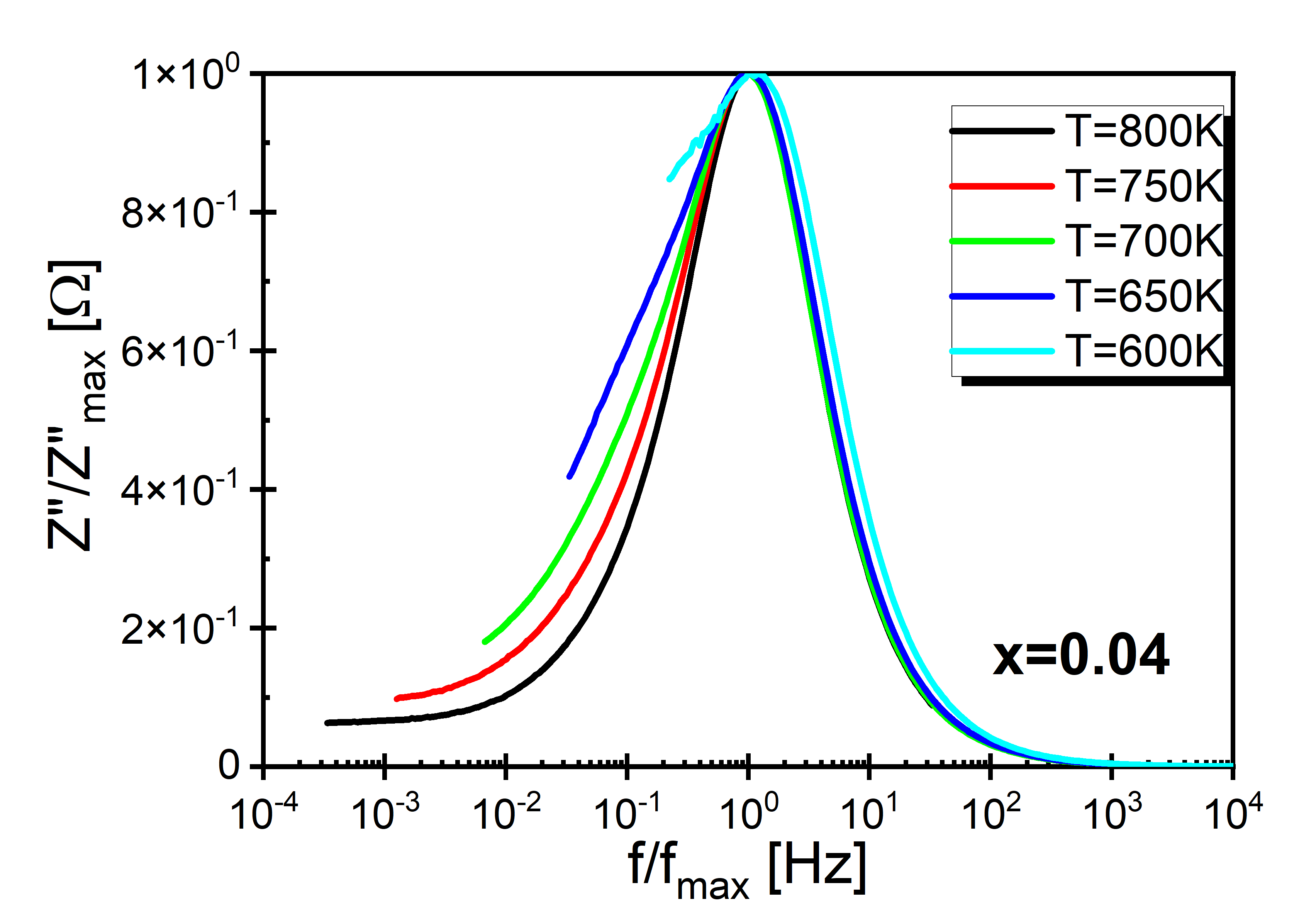

Supplement: Supplementary file 1 [file materials-17-04360-s001.zip › Figure S1a.png]

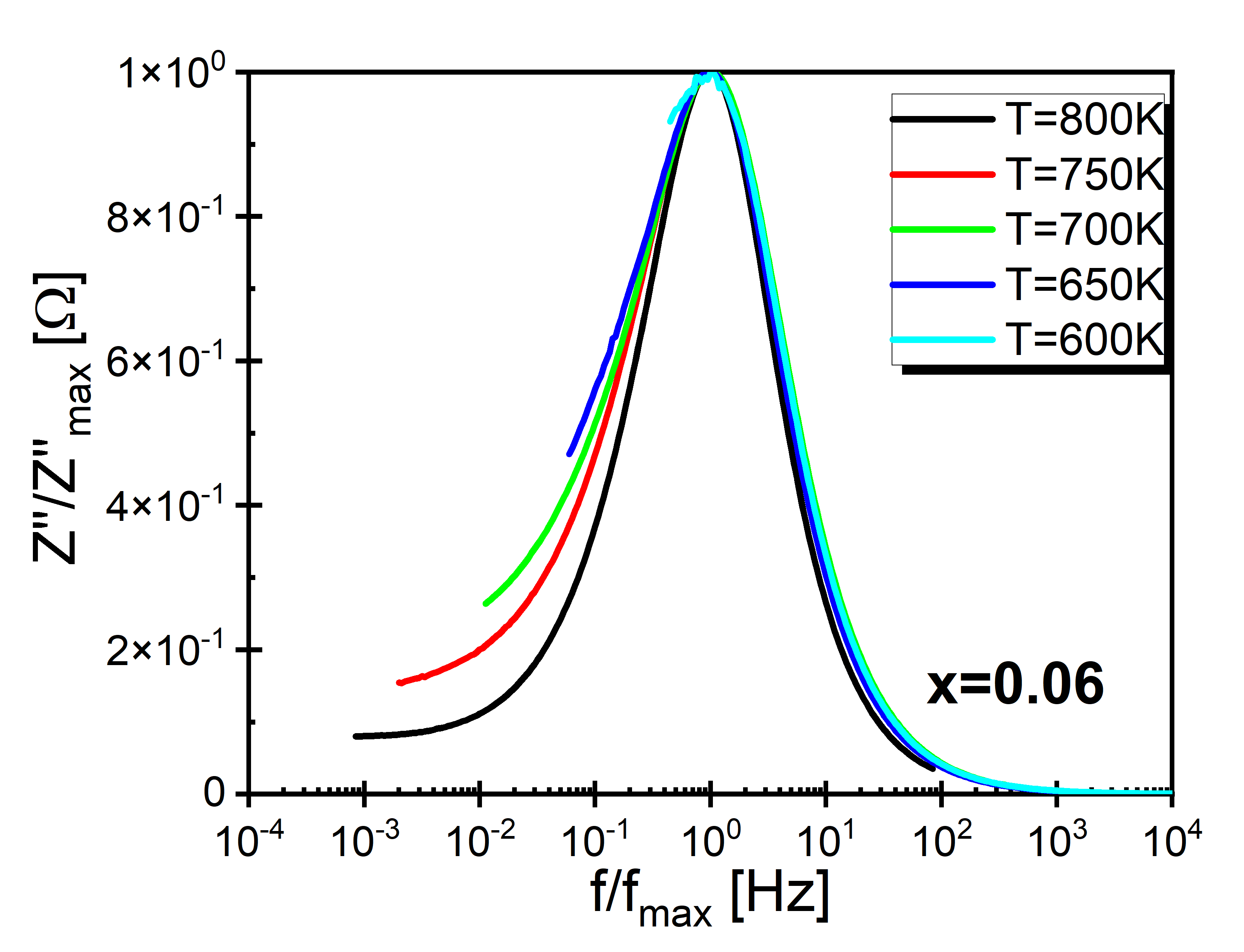

Supplement: Supplementary file 1 [file materials-17-04360-s001.zip › Figure S1b.png]

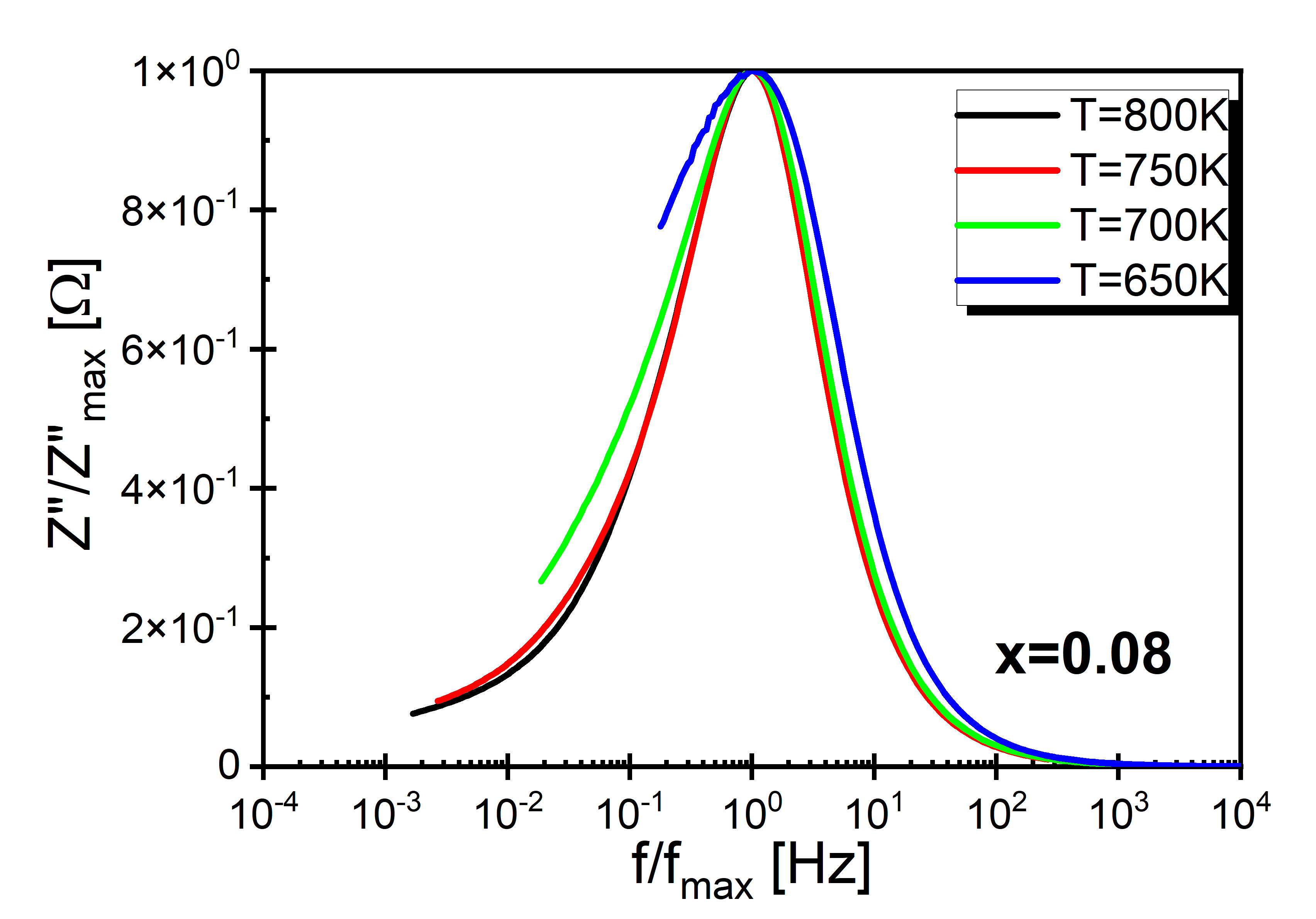

Supplement: Supplementary file 1 [file materials-17-04360-s001.zip › Figure S1c.png]

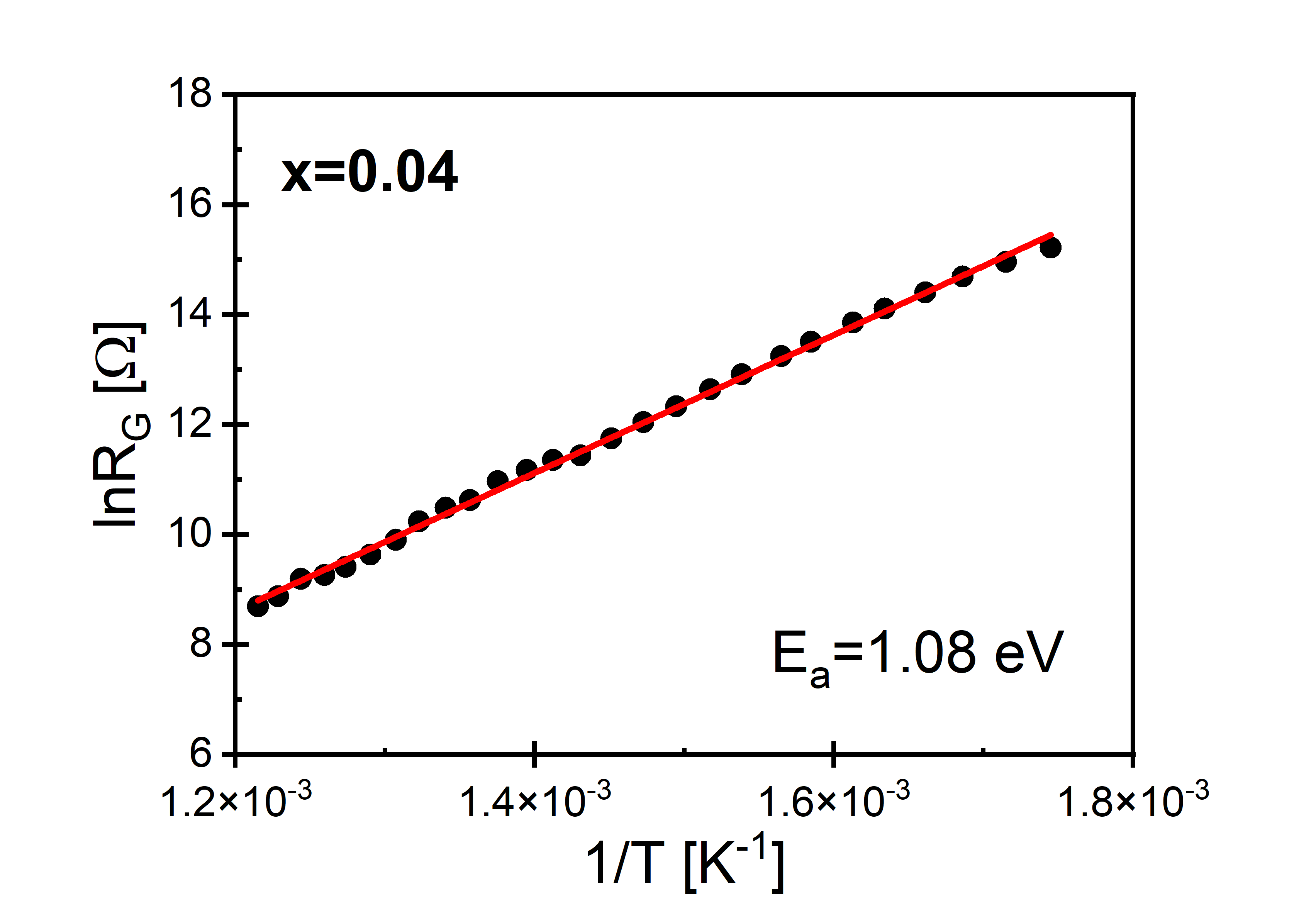

Supplement: Supplementary file 1 [file materials-17-04360-s001.zip › Figure S2a.png]

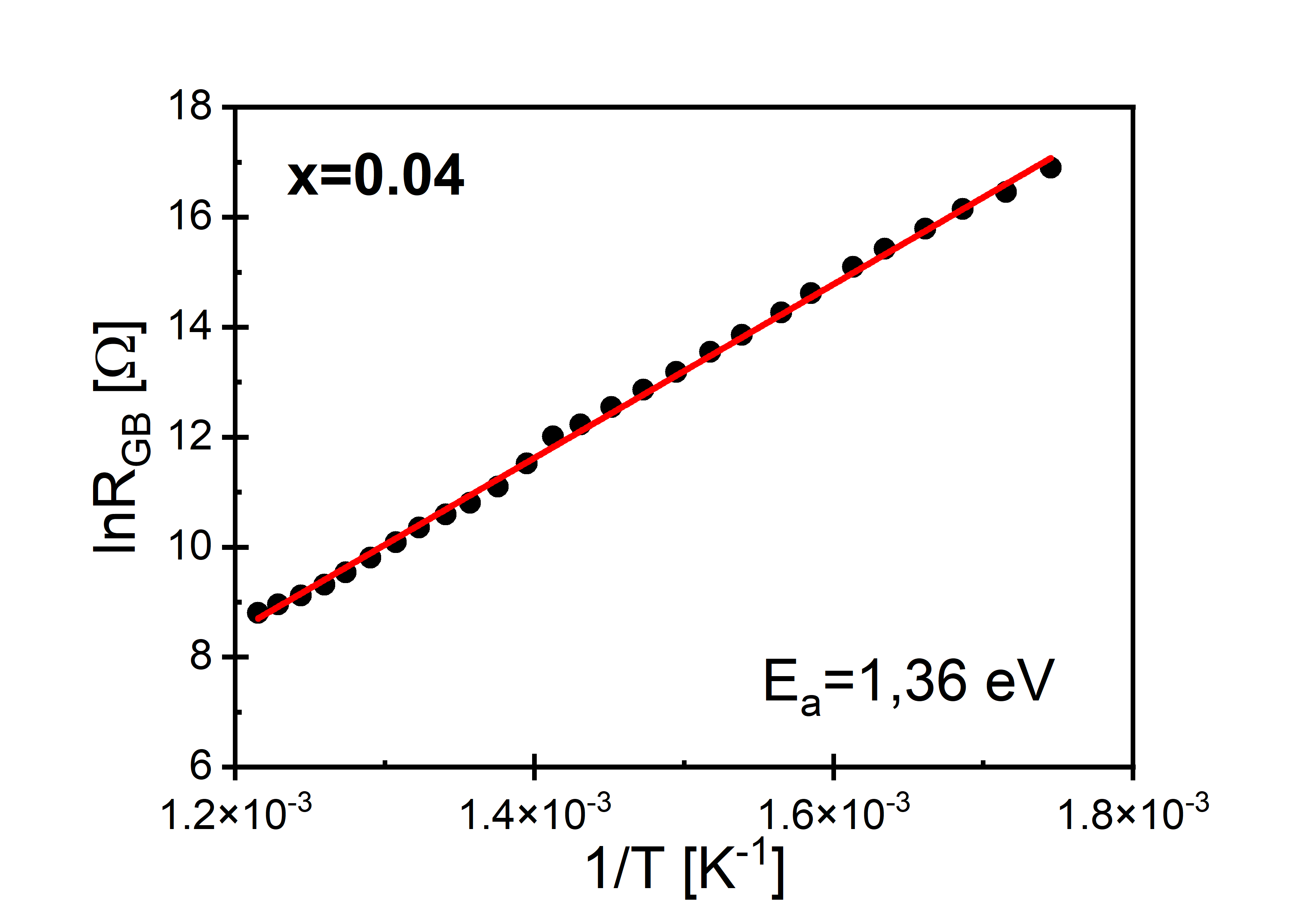

Supplement: Supplementary file 1 [file materials-17-04360-s001.zip › Figure S2b.png]

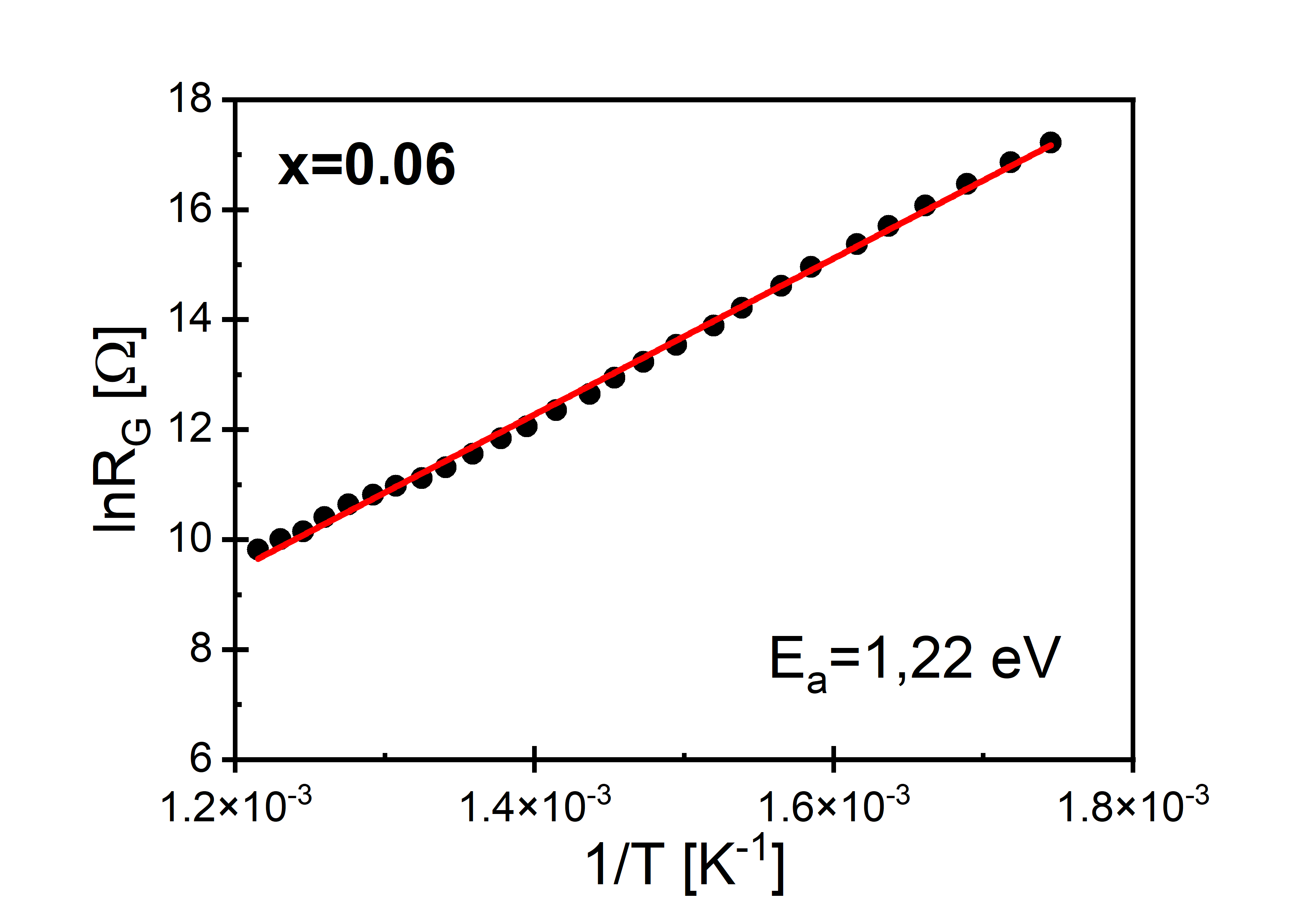

Supplement: Supplementary file 1 [file materials-17-04360-s001.zip › Figure S2c.png]

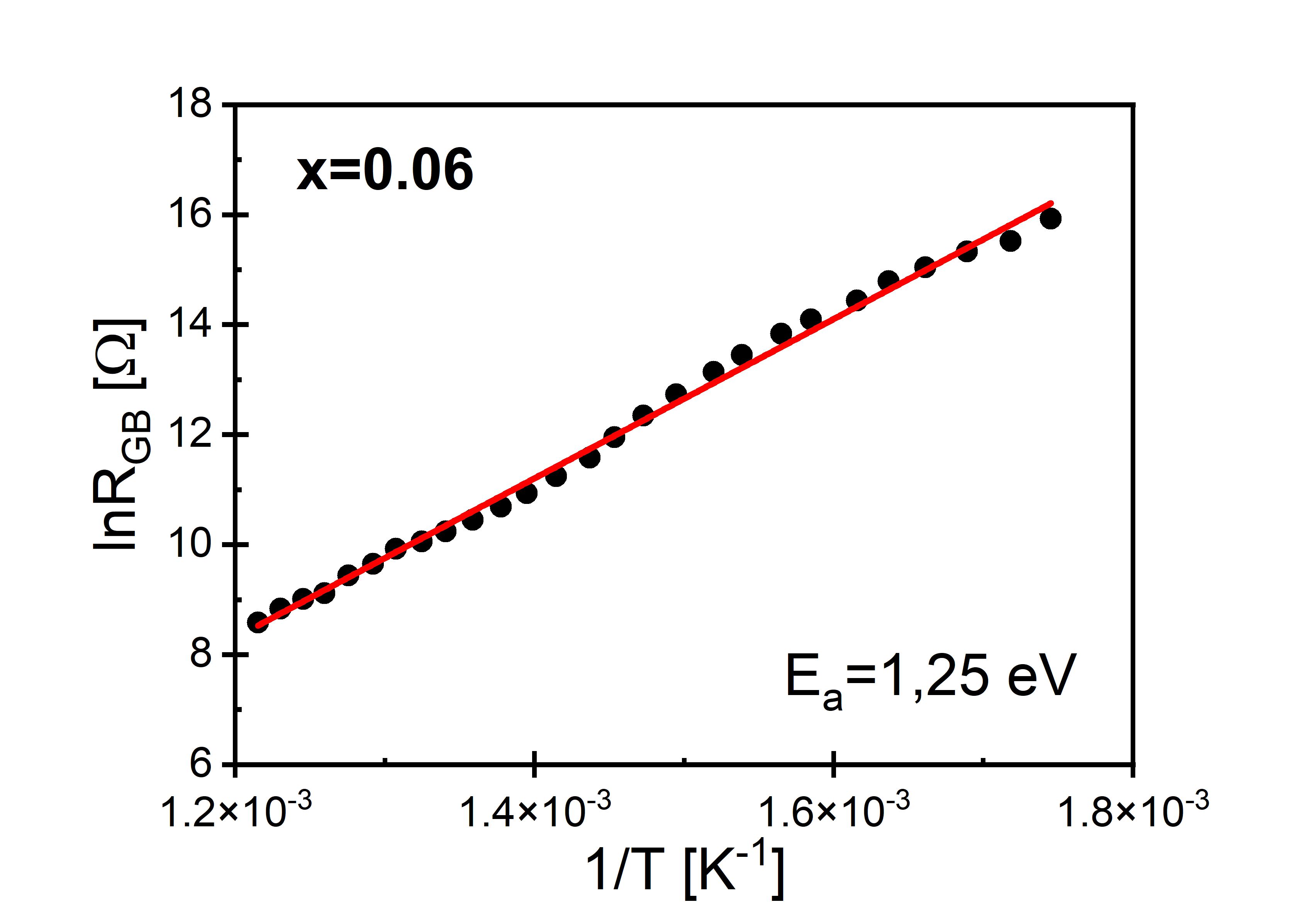

Supplement: Supplementary file 1 [file materials-17-04360-s001.zip › Figure S2d.png]

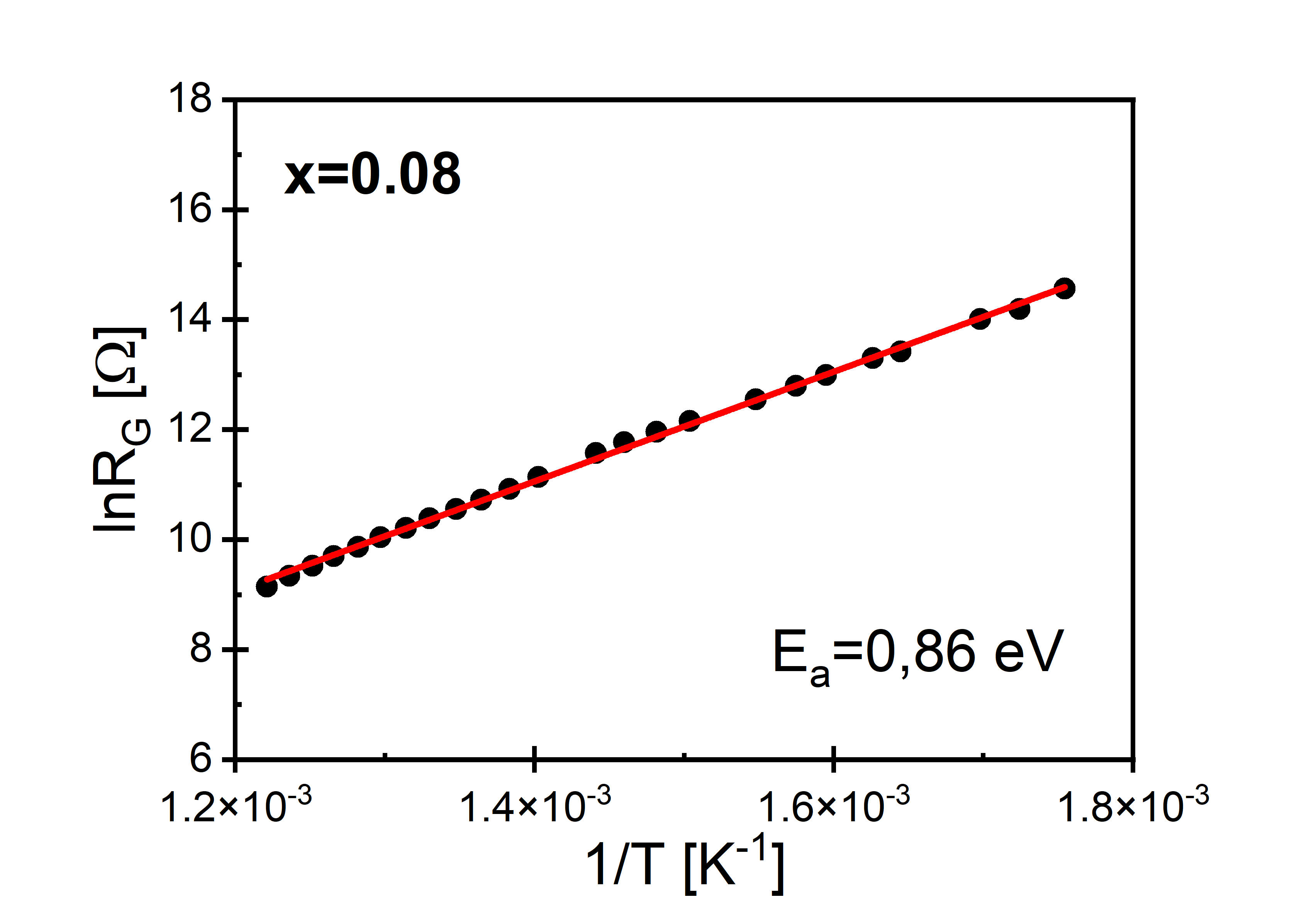

Supplement: Supplementary file 1 [file materials-17-04360-s001.zip › Figure S2e.png]

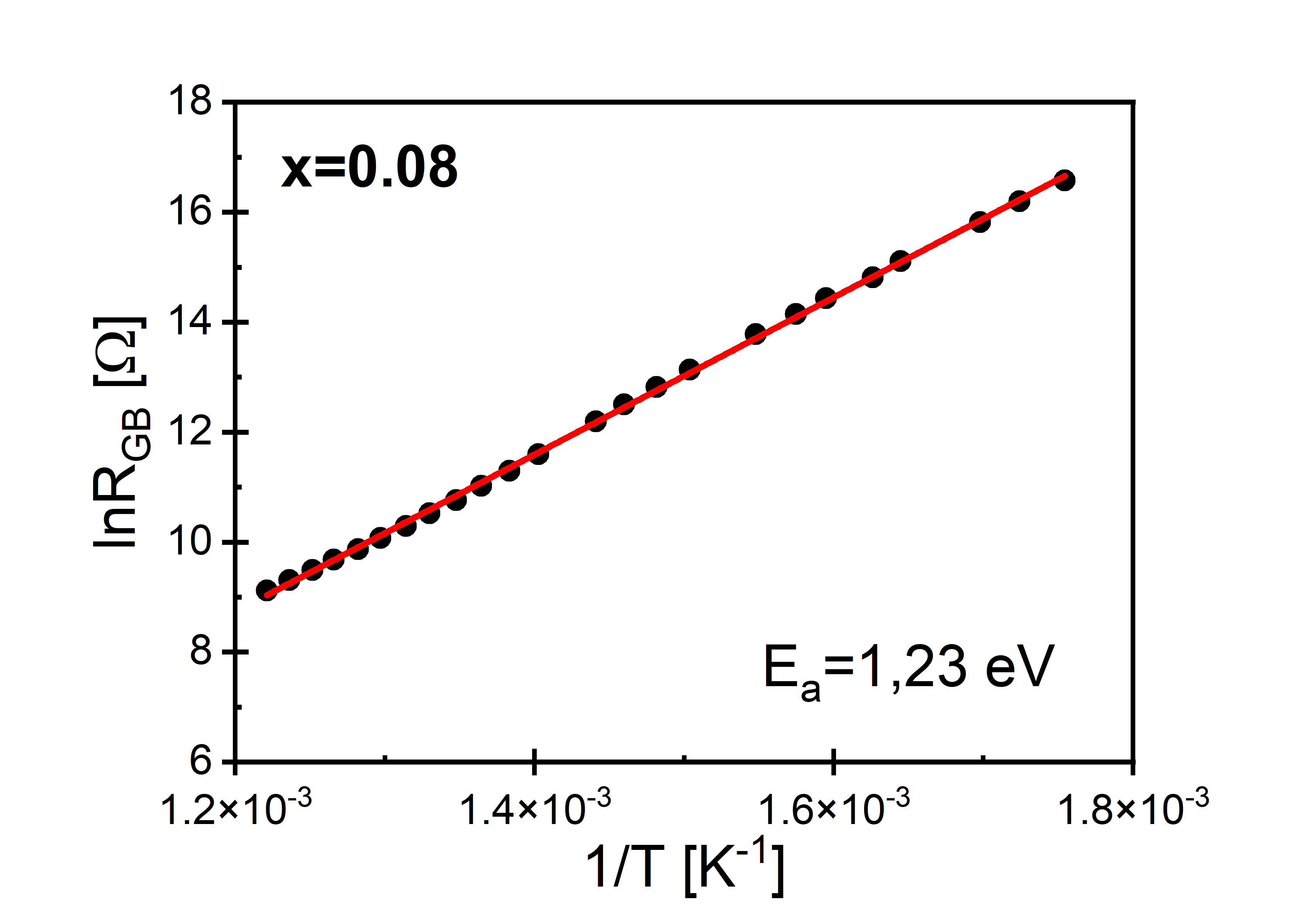

Supplement: Supplementary file 1 [file materials-17-04360-s001.zip › Figure S2f.png]

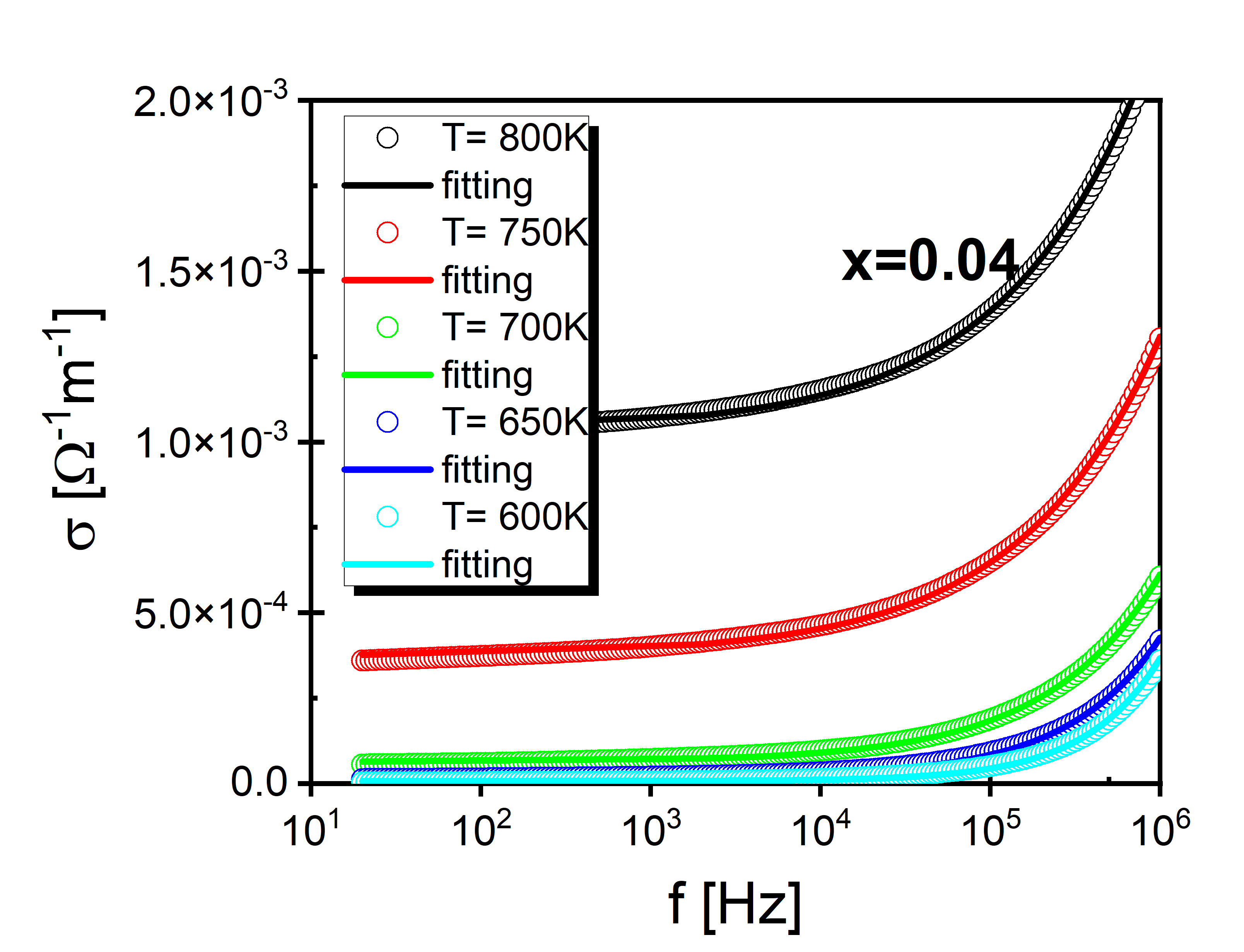

Supplement: Supplementary file 1 [file materials-17-04360-s001.zip › Figure S3a.png]

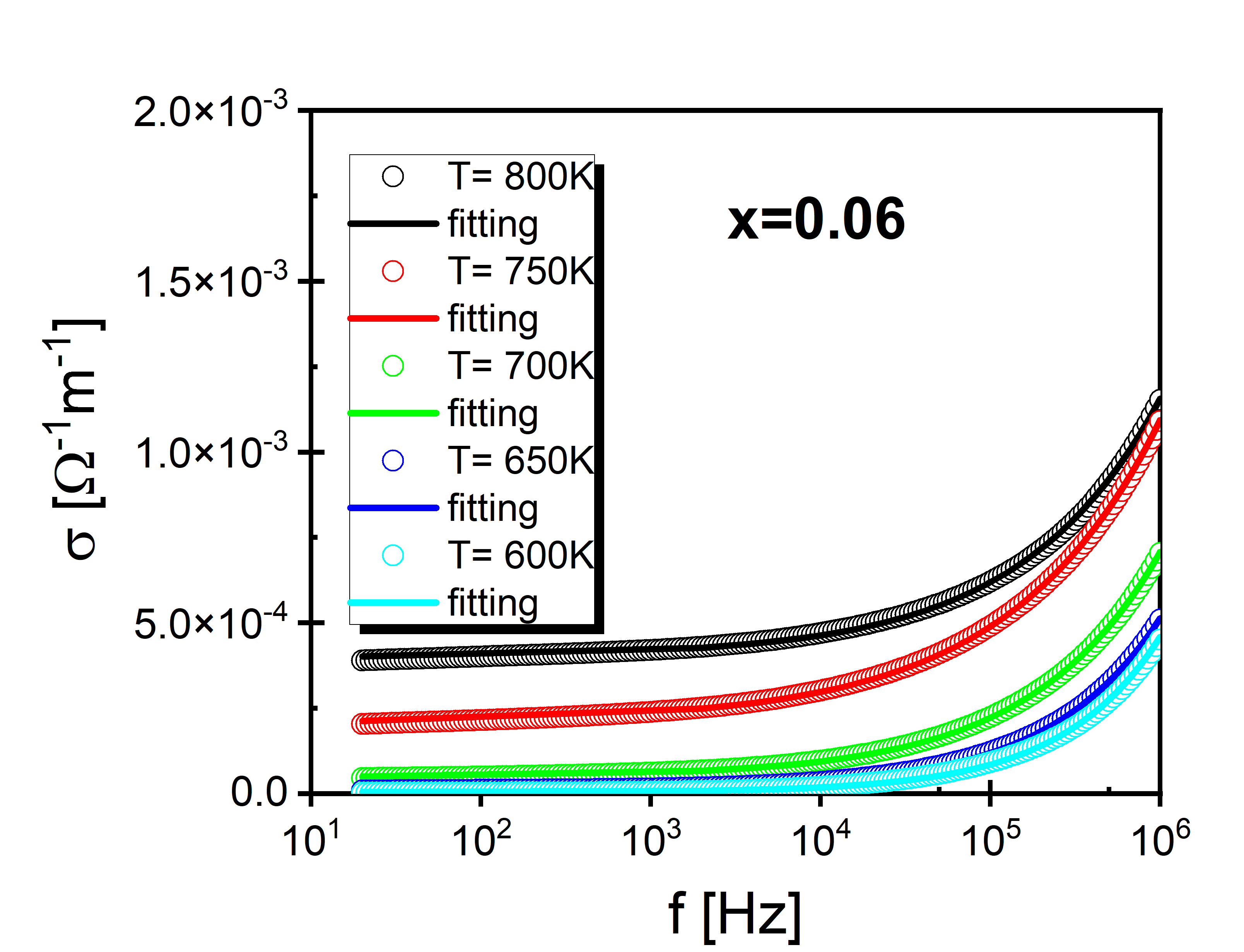

Supplement: Supplementary file 1 [file materials-17-04360-s001.zip › Figure S3b.png]

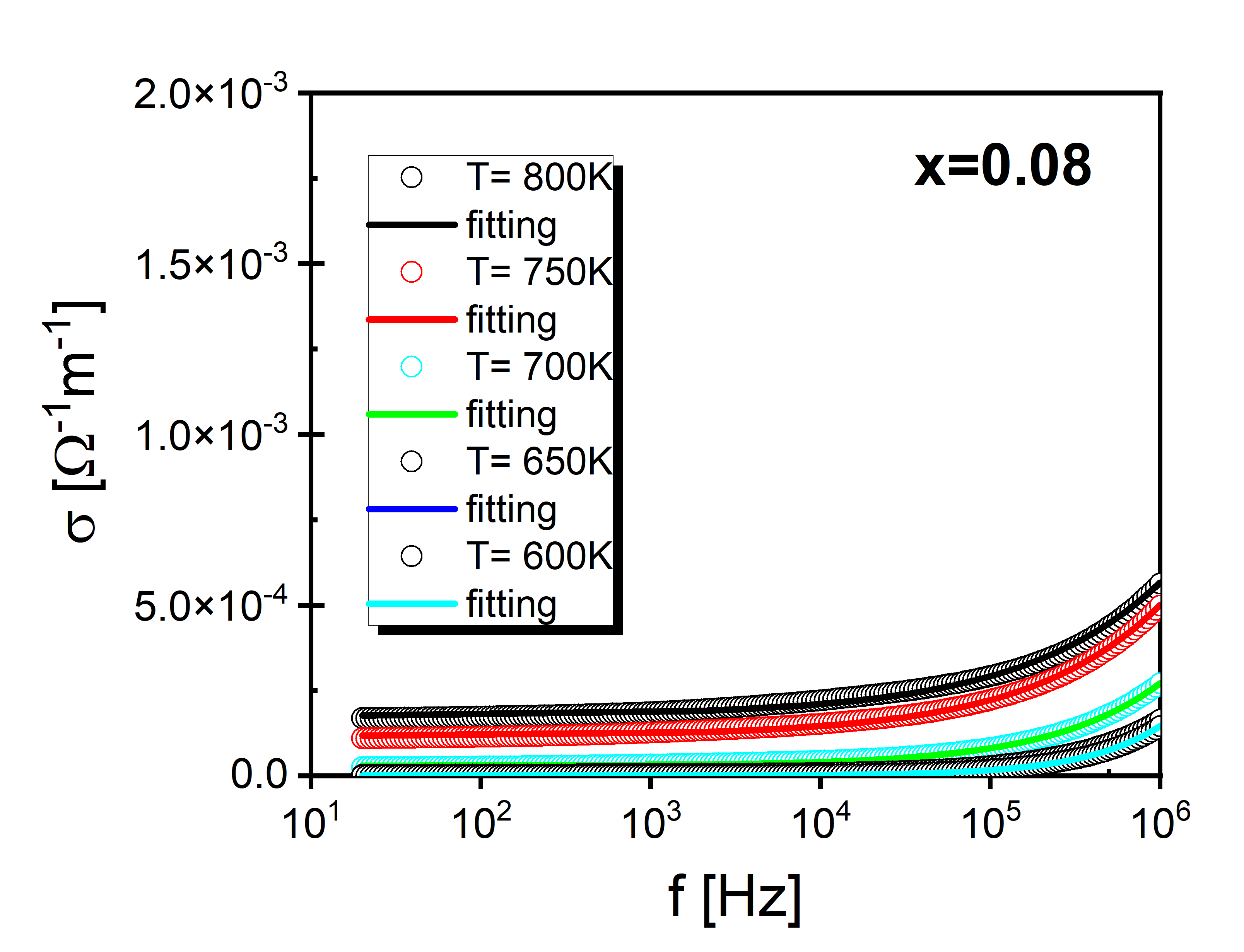

Supplement: Supplementary file 1 [file materials-17-04360-s001.zip › Figure S3c.png]

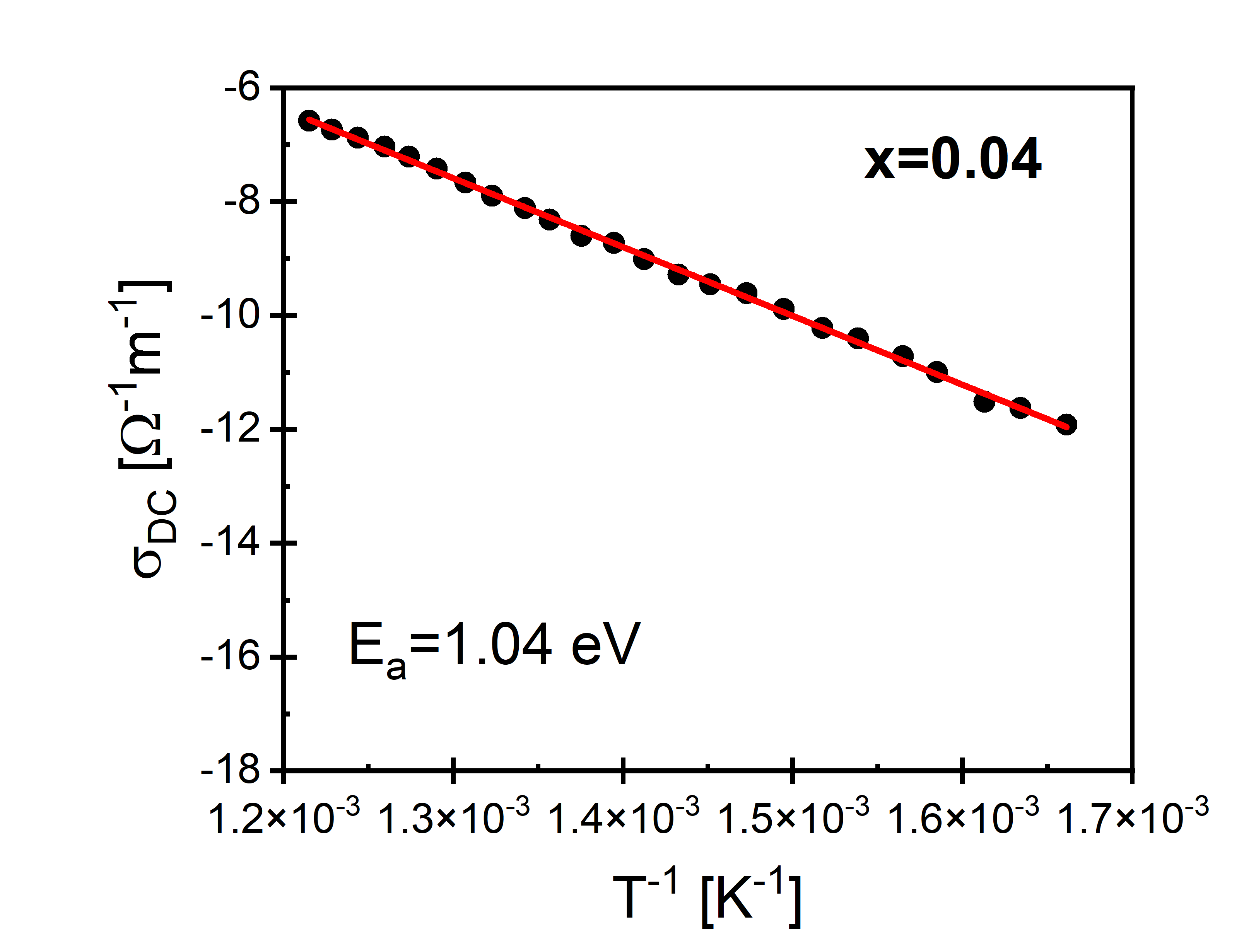

Supplement: Supplementary file 1 [file materials-17-04360-s001.zip › Figure S4a.png]

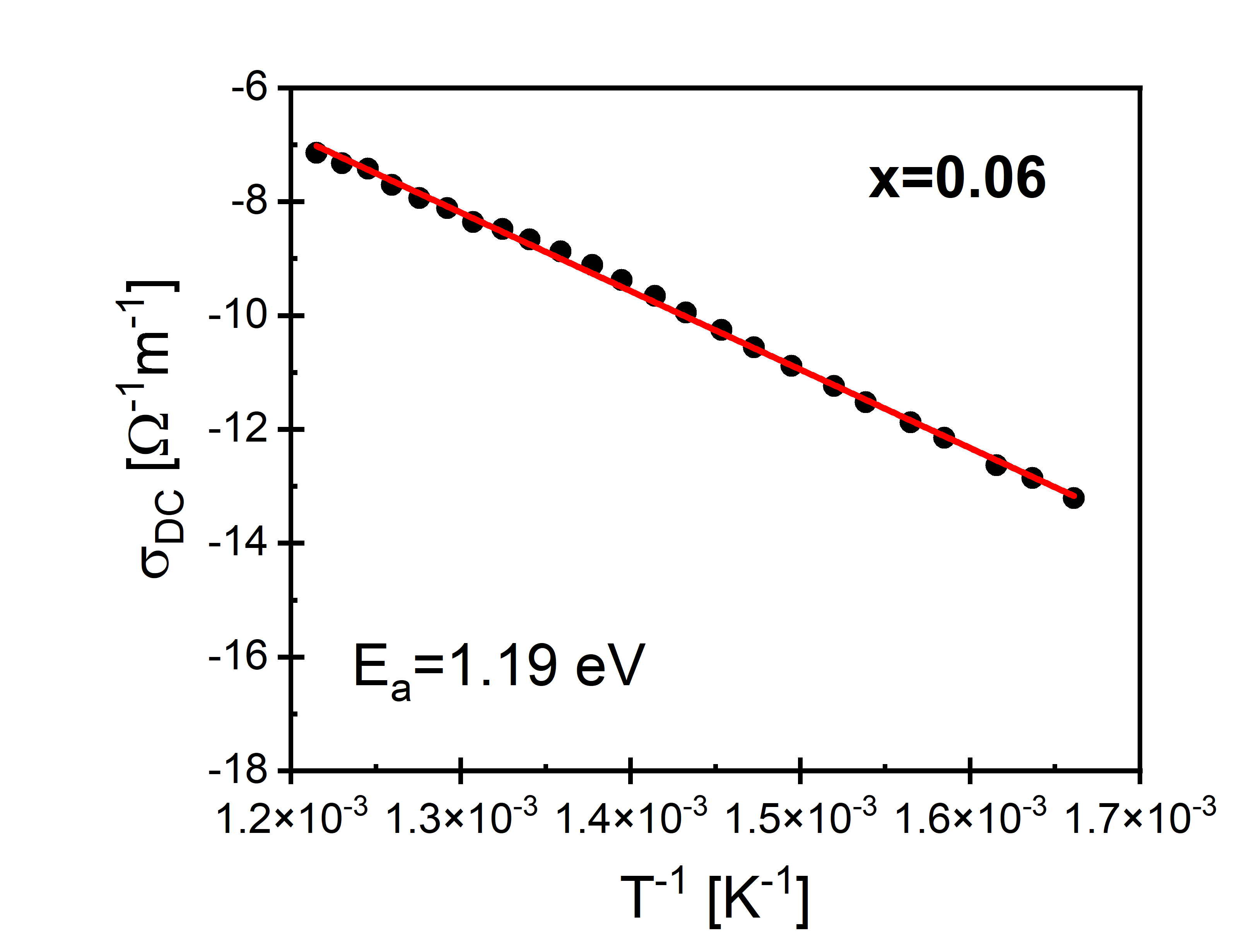

Supplement: Supplementary file 1 [file materials-17-04360-s001.zip › Figure S4b.png]

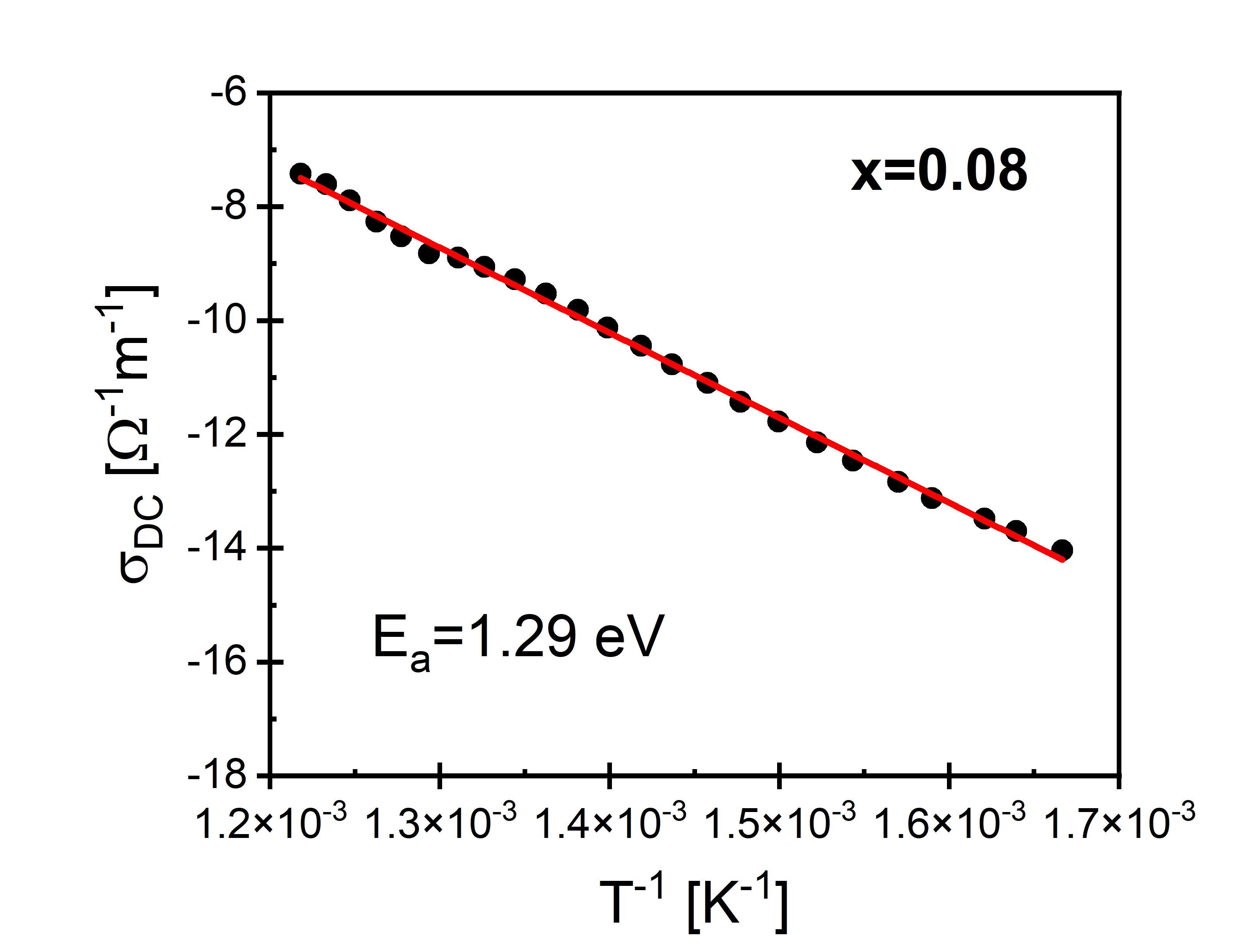

Supplement: Supplementary file 1 [file materials-17-04360-s001.zip › Figure S4c.png]
